# Involvement of TSP1 and MMP9/NGAL in Angiogenesis during Orthodontic Periodontal Remodeling

**DOI:** 10.1155/2014/421029

**Published:** 2014-05-20

**Authors:** Petra Surlin, Isabela Silosi, Anne Marie Rauten, Manole Cojocaru, Lili Foia

**Affiliations:** ^1^Department of Periodontology, University of Medicine and Pharmacy of Craiova, 2 Petru Rares Street, 200349 Craiova, Romania; ^2^Department of Immunology, University of Medicine and Pharmacy of Craiova, 2 Petru Rares Street, 200349 Craiova, Romania; ^3^Department of Orthodontics, University of Medicine and Pharmacy of Craiova, 2 Petru Rares Street, 200349 Craiova, Romania; ^4^Department of Physiology, University of Medicine Titu Maiorescu of Bucharest, 187 Calea Vacaresti Street, 040051 Bucharest, Romania; ^5^Department of Biochemistry, University of Medicine and Pharmacy “Gr. T. Popa” Iasi, 16 Universitatii Street, 700115 Iasi, Romania

## Abstract

In the present study the aim was to measure the levels of Thrombospondin-1 (TSP1) and Lipocalin-2/matrix metalloproteinase 9 (MMP9/NGAL) complex in gingival crevicular fluid (GCF) at different time points of orthodontic treatment, to determine the relationship between these values and those of total-matrix metalloproteinase 9 (MMP9) and theirs implication in angiogenesis balance, in the situation of a good control of the bacterial plaque, emphasizing the role of TSP1 and MMP9/NGAL complex. GCF samples were collected from 16 young orthodontic patients requiring upper canine distalization (test tooth) with first premolar extraction. The contralateral canine (control tooth) was free from orthodontic force. For the orthodontic appliance, brackets Roth 0.018 inch with 0.012 inch NiTi archwire and a laceback were used. TSP1, MMP9/NGAL, and MMP9 increased from 1 hour before activation of orthodontic appliance to a maximum at 8 hours for MMP9 and 72 hours for MMP9/NGAL and TSP1. The results show a change in time of TSP1, MMP9/NGAL, and MMP9 levels in GCF of patients with this method of orthodontic treatment. The powerful correlation of MMP9/NGAL with TSP1 suggests their stronger involvement in angiogenesis processes in PDL during orthodontic periodontal remodeling, in the situation of a healthy periodontium and a good control of the bacterial plaque.

## 1. Introduction

Orthodontic forces generate a biologic response from periodontium components of a physiologic and biochemical nature. During the initial phase of orthodontic treatment, a cascade of events is produced: osseous remodeling, angiogenesis and aseptic inflammation. A complex chain of mediators, that modulates the aseptic inflammation, is released in the gingival crevicular fluid (GCF), as a response to the mechanical stress induced by orthodontic forces in the context of a good control of the bacterial plaque [1]. The blood vessels, one of the constituents of the periodontal ligament (PDL), are actively involved in orthodontic periodontal remodeling, by variation of their number, caliber, and permeability [2–4].

Angiogenesis is represented by the formation of new blood vessels and is the result of a balance between the proangiogenic and antiangiogenic molecules [5]. Processes of angiogenesis are favorized by the degradation of extracellular matrix (ECM), that serves also as a support for the newly formed blood vessels. Angiogenesis and infectious inflammation are two intricated processes with common mediators [6–8], so, a very good hygiene, performed at home and professionally, frequently checked and motivated, is very important to maintain the net of biomarkers expressed during initial orthodontic treatment in the limits of aseptic inflammation.

MMP9 (matrix metalloproteinase 9) or gelatinase B is an endopeptidase with multiple weight forms (especially proform of 92 kDa and active form of 82 kDa), known as an angiogenesis promoter [9, 10] that degrades gelatine, fibronectin, elastin, and type IV, V, VII, and X and the denatured type I collagen. Recently, there were determined MMP9 concentrations in the gingival crevicular fluid (GCF) during the orthodontic periodontal remodeling, at different time intervals in a period of 180 days but using another method of detection other than ELISA [11].

NGAL or Lipocalin-2 is a small protein that may exist as a 25 kDa monomer, dimer, or heteromer in complex with MMP9. The MMP9/NGAL complex was revealed in systemic diseases and in GCF of patients with a poor control of the bacterial plaque with periodontal disease [12] but not during orthodontic treatment. It is a heavy weight protein (130 kDa), in which NGAL could have the role of protecting the molecules of MMP9 against their degrading [13].

TSP1 (Thrombospondin-1) is a glycoprotein, one of the endogenous inhibitors of angiogenesis, its main antiangiogenic site is type I collagen repeats and is active both as whole molecule and as fragments [9, 14–16]. Implication of TSP1 in inhibiting angiogenesis is by direct mechanism through interaction with specific receptors and indirect by influencing the activity of other mediators of angiogenesis [5]. So, TSP1 suppresses the mobilization of VEGF and the activation of proMMP9 [14, 17]. On the other hand, the increased levels of TSP1 lead to the stimulation of in vitro MMP9 production from endothelial cells in remodeling tissues with required degrading of the ECM [17]. There is no study in the literature about the levels of TSP1 in GCF or the role of TSP1 in orthodontic periodontal remodeling.

The present study aimed to measure the levels of TSP1, MMP9/NGAL, and total MMP9 in GCF 1 h before and after 1, 4, 8, 24, and 72 hours, 1 and 2 weeks from the orthodontic activation in the situation of a good control of the bacterial plaque, to determine the relationship between them and their implication in angiogenesis balance in PDL especially for MMP9/NGAL and TSP1.

## 2. Materials and Methods

The study was approved by the Ethics Committee of the University of Medicine and Pharmacy from Craiova, Romania, and informed consent of patients' tutors was obtained.

### 2.1. Subjects

The patients were enrolled according to the following criteria: (1) patients in general good health, (2) with a good oral hygiene, (3) with clinically and radiological healthy periodontal tissues, (4) no therapy with antibiotics in the last 3 months, and (5) no use of anti-inflammatory drugs in the previous 30 days. One week before sampling, the patients underwent a session of scaling and motivation for oral hygiene. GCF samples were collected from 16 orthodontic young patients requiring upper canine distalization with first premolar extraction (10 females and 6 males, age range 13 to 17 years, mean age 14 ± 1.67 years). For the orthodontic appliance, there are placed brackets Roth 0.018 inch (GAC Intl, Bohemia, USA) with 0.012 inch NiTi archwire (GAC Intl, Bohemia, USA) and a laceback made from 0.010 inch stainless wire, placed and activated 21 days after the premolar extraction. This appliance has a lesser rate of canine displacement but a more controlled canine movement was obtained [18].

### 2.2. GCF Sampling

GCF samples were collected using a technique previously described [19] and prior to any other clinical procedures to avoid blood contamination. The samples were taken from the distal side of an upper canine requiring distalization on which was placed a laceback appliance, considered as test tooth (TT) and the contralateral canine considered as control tooth (CT) on which no force was applied. The tooth was isolated with cotton rolls and the minimal supragingival plaque was removed using a curette (HU-Friedy, USA), without touching the marginal gingiva. The sites were gently dried with air syringe and saliva ejector was put in place to avoid salivary contamination. The GCF was collected with paper strips (Oraflow, USA) inserted into the distobuccal crevice until mild resistance was felt and left there for 30 sec. The 3 samples were taken at 1-minute intervals and used to determine the levels of MMP9, MMP9/NGAL, and TSP1 at each time point in this order. The same technique was used for both test and control tooth. The absorbed GCF was measured with a precalibrated device (Periotron 8000, Oraflow, USA); then it was eluted from the paper strips in 100 *μ*L PBS (phosphate-buffered saline) pH = 7.2. The eluted samples were stored in polypropylene tubes at −20°C prior to analysis.

### 2.3. Assay of GCF Levels of MMP9/NGAL, MMP9, and TSP1

The GCF levels of the three substances were determined using commercial ELISA kits for MMP9, MMP9/NGAL, and TSP1 (Quantikine, R&D Systems, Minneapolis, USA) and the results were expressed in ng/mL. The kit used for total MMP9 measurement detects 82 and 92 kDa forms, while the kit for MMP9/NGAL detects 130 kDa complex between another form of MMP9 and Lipocalin-2 (NGAL).

Every kit component was used according to the manufacturer's indications. The absorbance was measured at 450 nm with a correction at 540 nm to reduce optical imperfections on the reading plate.

### 2.4. Statistical Methods

We used the mean ± standard deviation (SD) to express the levels of the 3 substances. After Friedman's test, Wilcoxon test was also performed to compare the means within each group and Mann-Whitney *U* test to compare the means between groups (*P* < 0.05 for significantly statistical differences) and Pearson's test for statistical correlations was performed using professional software (SPSS 16.0, Chicago, USA).

## 3. Results

Throughout the study, the periodontal health was very good, the patients kept a good oral hygiene, and at each presentation the patients were motivated to maintain it; the accumulation of plaque was minimal. There was not any indication for antibiotic and anti-inflammatory therapy, and therefore there were no patients excluded from the study.

### 3.1. Levels of MMP9, MMP9/NGAL, and TSP1 in GCF

The results obtained for the TT showed an increase of GCF levels of MMP9 from 1 hour before the orthodontic appliance (−1 h as baseline level) to a maximum at 8 hours (approximately 1.6-fold compared to the baseline level), followed by a decrease reaching the value of baseline at 72 hours and stabilization starting with first week at levels higher than baseline.

Statistically significant differences (*P* = 0.01) were found between mean levels of MMP9 at 4, 8, 1, and 2 weeks and the baseline level. There was no statistically significant difference between the levels at 1, 24, and 72 hours and baseline.

For the MMP9/NGAL in GCF of TT, the values remained at the baseline level in the first 4 hours followed by an increase with maximum level at 72 hours (approximately 1.5-fold compared to the baseline level) and returned to the initial measured level in the first week. Statistically significant differences (*P* = 0.002) were found between the levels of MMP9/NGAL in GCF at 8, 24, and 72 hours and baseline. No significant difference was found between the values at 1 and 4 hours, 1 week, 2 weeks, and baseline.

The results for TT showed that GCF levels of TSP1 remained at the baseline values in the first 4 hours and began to increase from 8 hours after to a maximum at 72 hours (approximately 1.5-fold compared to the baseline level). In the first week, the level returned to the baseline. Statistically significant differences (*P* = 0.05) were found between the levels of TSP1 in GCF at 24 and 72 hours and baseline. No significant difference was found between the values at 1, 4, and 8 hours, 1 week, 2 weeks, and baseline.

**Figure 1:**
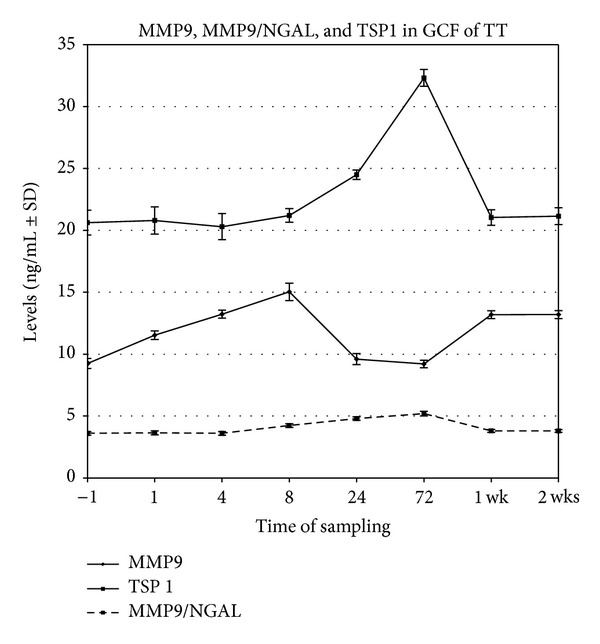
Evolution in time (*x*-axis) of GCF levels ± SD (*y*-axis) of MMP9/NGAL, TSP1, and MMP9 in TT.

The dynamics in time of MMP9, MMP9/NGAL, and TSP1 in GCF of TT is shown in Figure 1.

### 3.2. Correlations between Levels of MMP9, MMP9/NGAL, and TSP1 at Different Time Points of Orthodontic Treatment

**Figure 2:**
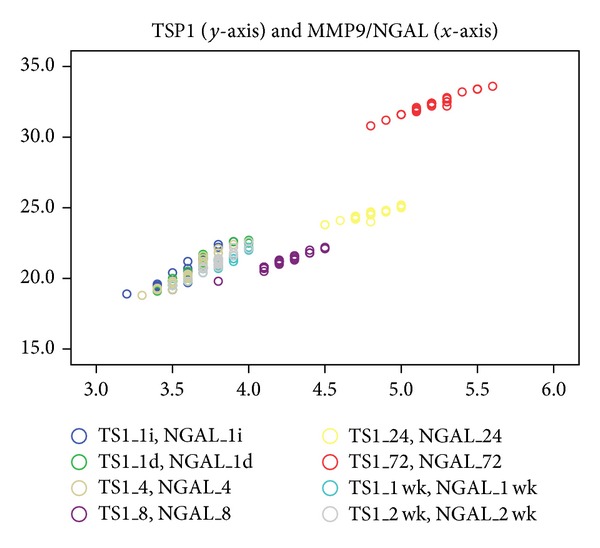
Strong and significant correlations were found between MMP9/NGAL (*x*-axis) and TSP1 levels (*y*-axis) in all samples in every moment of the study in TT (*r* varied between 0.931 and 0.981, *P* = 0.0001) (legend: 1i: 1 hour before, 1d: 1 hour after, wk: week).

Strong and significant correlations were found between MMP9/NGAL and TSP1 levels in all samples in TT (*r* varied between 0.931 and 0.981, *P* = 0.0001) (Figure 2).

**Figure 3:**
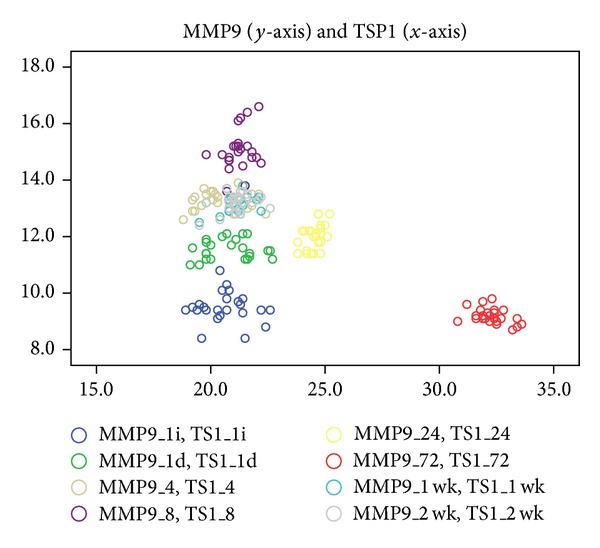
Poor and not significant correlations between MMP9 (*y*-axis) and TSP1 levels (*x*-axis) at 1 (*r* = 0.089, *P* = 0.68), 4 (*r* = 0.049, *P* = 0.8), and 8 hours (*r* = 0.268, *P* = 0.2) and 1 week (*r* = 0.359, *P* = 0.08) and 2 weeks (*r* = 0.349, *P* = 0.09) and moderate but in inverse significant relation at 24 and 72 hours (*r* = −0.405, *P* = 0.05) after activation of orthodontic treatment for TT (legend: 1i: 1 hour before, 1d: 1 hour after, wk: week).

Between MMP9 and TSP1 levels the correlations are poor and not significant at 1, 4, and 8 hours, 1 week, and 2 weeks and moderate but in inverse relation at 24 and 72 hours after activation of orthodontic appliances (Figure 3).

**Figure 4:**
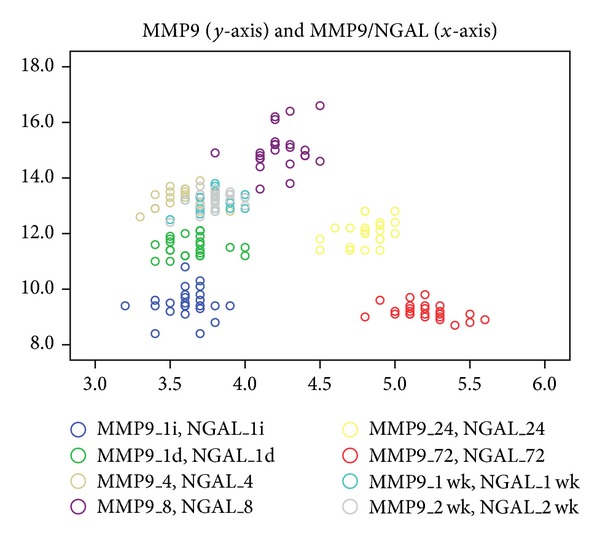
Poor and not significant correlations between MMP9 (*y*-axis) and MMP9/NGAL levels (*x*-axis) in TT in every moment of the study (*r* varied between 0.042 and 0.381, *P* > 0.05) except the 72-hour levels when the correlation was moderate, significant but inverse (*r* = −0.418, *P* = 0.04) (legend: 1i: 1 hour before, 1d: 1 hour after, wk: week).

MMP9 and MMP9/NGAL levels in TT were poorly correlated without statistically significance in every moment of the study (*r* varied between 0.042 and 0.381, *P* > 0.05) except the levels at 72 hours when the correlation was moderate but inverse and significant (Figure 4).

For the CT, GCF levels of MMP9, MMP9/NGAL, and TSP1 at any time point did not differ statistically from their baseline levels in TT. There were no statistically significant differences between volumes of GCF samples neither for TT or CT; (data not shown). Because of the small group of patients correlations between subgroups of sexes were not performed.

## 4. Discussions

Being an exudate that reflects the events in the periodontium, GCF may be used to detect the levels of certain biologically active substances. There are studies upon the expression of biomarkers in GCF during different types of orthodontic tooth movements including matrix metalloproteinases (MMPs)—which include MMP9 [11, 20–22]. There is no study on the 130 kDa MMP9/NGAL complex in GCF in orthodontic patients or on TSP1. There are scientific proofs regarding the involvement of these biomarkers in angiogenesis in oral wound healing [8], but studies searching forms of MMP9 involved in angiogenesis during orthodontic movement with a good control of the bacterial plaque and regarding the relationship between the levels in GCF of these forms and TSP1 are not available.

Concerning the angiogenesis in PDL of patients in orthodontic treatment, VEGF expression was studied the most. This is recognized as one of the main regulators of angiogenesis [23] either by increasing blood vessels permeability [24] or monocyte chemotaxis [25]. The interrelations between VEGF and TSP1 were studied but not clarified yet [26]. During orthodontic treatment, VEGF expression was related to the osteoclast activity in the tension side. An increase in level of the osteoblasts in which the VEGF was highly expressed was revealed at 10 days from the onset of treatment [27]. A combination of (rh) VEGF injection and orthodontic force may lead to a higher rate of tooth displacement [28]. Thus, it was shown [29] that VEGF plays an important role in periodontal remodeling during orthodontic tooth movement.

One of the mechanisms through which TSP1 regulates angiogenesis is linked to the suppression of the MMP9 expression from endothelial cells [17, 30], but neither the molecular forms implicated nor their dynamic were investigated.

Different forms of MMP9 were detected in GCF of patients with orthodontic devices after long term treatment [22] or during initial phase [11]. In the study of Bildt et al. [22] gelatin zymography and Western blotting were used as techniques to reveal forms of MMP9 in GCF. The mostly found forms were of 92 and 82 kDa weight, their presence was presumed to be associated with their gelatinolytic activity in ECM. A maximum level of MMP9 was also found [11], using multiplexed bead immunoassay, 1 hour after force appliance followed by a fast decrease in the first 24 hours and a slow increase in the next 4 weeks.

In the present study, all patients had a good oral hygiene with a good control of plaque across entire study. The measurements performed at short time period during the first 24 hours show that the maximum level of total MMP9 (92 and 82 kDa) detected by the kit used was reached at 8 hours after a continuous increase. The level of total MMP9 starts to decrease, the minimal levels were reached at 72 hours when the baseline level was attained and remained higher two weeks afterwards. The difference between these results and those in the aforementioned study are probably due to the technique of MMP9 detection and the forms identified by each method.

MMP9/NGAL complex was revealed in GCF of patients with periodontal disease [12] and accumulation of bacterial plaque as having a stronger expression and correlation with myeloperoxidase (MPO) than 92 and 82 kDa forms of MMP9. Also, in the same study, there were stronger correlations in patients in periodontal health than in those with chronic periodontitis between the total MMP9 and MMP9/NGAL complex, with authors attributing it to the fact that most of MMP forms found in GCF of periodontal healthy patients come from PMNs, while in patients with periodontal disease they might be from other sources also [12]. Those data are in accordance with the present study in teeth with healthy periodontium not exposed to any orthodontic force—CT and baseline for TT. This high molecular weight MMP was revealed in the urine of cancer patients and the conclusion was that the role of NGAL is to protect the form of MMP9 involved in enzymatic degrading, thus supporting its activity [13].

In the present study, levels of MMP9/NGAL in GCF began to rise at 8 hours from the appliance, when total MMP9 reached the maximum. MMP9/NGAL continued to rise until 72 hours when reaching a maximum, while the MMP9 decreased from 8 at 72 hours, when reaching baseline. The pattern of MMP9/NGAL variation was different from the MMP9's, with which established a moderate but inverse correlation at 72 hours.

TSP1 was detected in PDL [31] together with other glycoproteins as vitronectin, fibronectin, and laminin but the dynamic of its expression in periodontal health and disease was not investigated yet. Results are sporadic and contradicting, so that the TSP1 role and its interactions with other biologic active substances in GCF are still not elucidated. The intensity of TSP1 expression was evaluated by immunohistochemistry (IHC) in the gingiva of patients with periodontitis without finding any significant differences with the healthy tissue [32]. Other authors, in a study focused on the relationship *ανβ*6 integrin-periodontitis, took into consideration the involvement of TSP1 in periodontal disease via MMPs and TGF*β*-1 [33], with the involvement of TSP1 in activation of TGF 1*β* [34, 35] being already shown.

In the present study, the pattern of TSP1 variation is similar to that of MMP9/NGAL with which established stronger and direct correlations in every moment of sampling, rather with total MMP9, suggesting that this form of presentation is actively involved in angiogenesis balance during orthodontic treatment, with the increase of MMP9/NGAL levels being associated with increase of TSP1 expression.

MMP9 forms of 92 and 82 kDa are more active in the first hours after orthodontic activation, reaching the maximum at the moment when TSP1 starts to increase, collagen remodeling in the extracellular matrix being necessary to the foreshadowing of new blood vessels.

It would be interesting to search in future the correlations between MMP9/NGAL, TSP1, and other factors involved in angiogenesis and aseptic inflammation to find the place occupied by these biomarkers in the mediators' fall that is activated during the initial phase of orthodontic treatment.

## 5. Conclusion

As a conclusion, this study shows a change in time of MMP9, MMP9/NGAL, and TSP1 levels in GCF of patients with orthodontic treatment for canine distalization and the powerful correlation of MMP9/NGAL with TSP1 suggests the strong involvement of MMP9/NGAL as a promoter of angiogenesis processes in PDL during orthodontic periodontal remodeling.
